# Genome Sequence of Cupriavidus campinensis Strain G5, a Member of a Bacterial Consortium Capable of Polyethylene Degradation

**DOI:** 10.1128/mra.00553-22

**Published:** 2022-09-20

**Authors:** Beate Schneider, Friedhelm Pfeiffer, Mike Dyall-Smith, Hans-Jörg Kunte

**Affiliations:** a Bundesanstalt für Materialforschung und -prüfung, Division of Biodeterioration and Reference Organisms, Berlin, Germany; b Computational Biology Group, Max Planck Institute of Biochemistry, Martinsried, Germany; c Veterinary Biosciences, Faculty of Veterinary and Agricultural Sciences, University of Melbourne, Parkville, Australia; University of Maryland School of Medicine

## Abstract

Nine different bacterial isolates were recovered from landfills. Each isolate was obtained in pure culture. As a consortium, the bacteria degrade polyethylene. The complete genome sequence of strain G5 was determined by PacBio sequencing. Using the TYGS for taxonomic classification, strain G5 was assigned to the species Cupriavidus campinensis.

## ANNOUNCEMENT

As part of a bacterial consortium, Cupriavidus campinensis G5 degrades polyethylene and was isolated from a former plastic landfill (Niemegk-Neuendorf, Germany [52°04′59″N, 12°41′59″E]) using a modified iChip procedure (1). A bacterial suspension from the landfill was diluted in soft agar, loaded into an iChip, and incubated for 4 weeks (2, 3). The emerging microcolony of strain G5 was streaked on agar, and cells from a single colony were inoculated into casein-soy broth (casein peptone, 15 g L^−1^; soymeal peptone, 5 g L^−1^; yeast extract, 0.5 g L^−1^; NaCl, 5 g L^−1^; glucose, 10 mM [pH 7.2]). After growth for 24 h at 30°C, cells were centrifuged, washed twice with phosphate-buffered saline, and kept at −80°C.

The following procedures were performed at Genewiz/Azenta (Leipzig, Germany), including DNA extraction using the Genomic-tip 100/G kit (Qiagen, Hilden, Germany), DNA shearing to ~10 kb using the g-TUBE device without size selection, quality (NanoDrop and pulsed-field gel electrophoresis analyses) and quantity (Qubit 2.0) assessments, and sequencing library preparation using the SMRTbell library preparation kit v2.0 (Pacific Biosciences [PacBio], Menlo Park, CA, USA). The library was sequenced on a PacBio Sequel sequencer (4) using one single-molecule real-time (SMRT) cell. The sequencing yielded 68,653 reads, from which 389,796 subreads were obtained (average length, 5,724 bp; read *N*_50_, 7,235 bp; total size, 2.231 Mbp). An assembly was performed within the SMRT Link Suite v5.0.0.6792 at Genewiz/Azenta, including checks for quality and coverage using default parameters. Subread coverage was reduced to 200-fold by random down-sampling, and the genome was automatically assembled using Canu v1.7 (5) with a coverage cutoff set to 40-fold and default parameters otherwise. The assembly was evaluated for overlapping contigs, which eventually were merged, and for potential assembly artifacts, which were dropped. Contigs were polished with raw subreads using Arrow v2.3.2 (https://github.com/PacificBiosciences/pbbioconda) with default parameters. Initial assembly generated 13 contigs, with a total length of 6,468,862 bp.

A supervised assembly using Geneious v10.2 (6) for mapping of PacBio reads to contigs, with subsequent integration and editing of contigs, was performed. Long duplications, such as two highly similar 27-kb prophages, were resolved by mapping adjacent unique regions to closely related genomes and by analyzing long PacBio reads; this resulted in two replicons. Chromosome 1 (GenBank accession number CP097330), which was obtained with 331-fold average coverage, is complete, circular, and 3,780,571 bp long, with a G+C content of 66.2%. Chromosome 2 (GenBank accession number CP097331), which was obtained with 339-fold average coverage, is complete, circular, and 2,524,655 bp long, with a G+C content of 66.7%. Start bases were set according to related chromosomes that are sufficiently similar and have been scrutinized for replication origin consensus sequences (7, 8). For GenBank accession number CP097330, the start base is consistent with that of GenBank accession number CP000352, upstream of *dnaA* (7). For GenBank accession number CP097331, the start base is consistent with that of GenBank accession number CU633750, upstream of *repA* (8). No additional replicons were detected.

The TYGS (9) assigned strain G5 to the species Cupriavidus campinensis, with a digital DNA-DNA hybridization (dDDH) value (d4) of 96.7% (95% confidence interval, 95.5 to 97.6%) (10). The small G+C content difference (0.20%) between the genome and the type strain, Cupriavidus campinensis LMG19282, supports the assignment. The genome was submitted to GenBank and annotated using PGAP v6.1 (11).

### Data availability.

The annotated genome has been deposited in GenBank under the BioProject accession number PRJNA837128 and the BioSample accession number SAMN28191058. The nucleotide sequence accession numbers are CP097330 (major chromosome) and CP097331 (minor chromosome). The Sequence Read Archive (SRA) accession number is SRR19184626.
